# The paradox of immunotherapy in NASH-HCC

**DOI:** 10.1038/s41392-021-00654-9

**Published:** 2021-06-10

**Authors:** Yao Peng, Chi Chun Wong, Jun Yu

**Affiliations:** 1 Department of Gastroenterology, Guangzhou First People’s Hospital, South China University of Technology, Guangzhou, China; 2 Institute of Digestive Disease and Department of Medicine and Therapeutics, State Key Laboratory of Digestive Disease, Li Ka Shing Institute of Health Sciences, CUHK-Shenzhen research Institute, The Chinese University of Hong Kong, Hong Kong, China

**Keywords:** Cancer, Immunology

A recent study published in Nature by Dominik Pfister et al.^1^ unveiled a darker side of immune checkpoint blockade (ICB) immunotherapy in the context of nonalcoholic steatohepatitis-associated hepatocellular carcinoma (NASH-HCC), whereby anti-PD1 treatment paradoxically accelerates hepatocarcinogenesis.

NASH is fast becoming the major liver disease in the developed world and is an emerging cause of HCC. Nevertheless, no specific drug is available for the treatment of NASH at present. ICB therapy, which aims to reinvigorate T cells, has profoundly shifted the paradigm for advanced HCC treatment in the past few years.^2^ ICB therapy primarily functions to activate cytotoxic CD8^+^ T cells whose activity is repressed in the tumor microenvironment (TME). As the key effectors of anti-tumor immunity, CD8^+^ T cells mediate immunosurveillance of premalignant hepatocytes, and depletion of CD8^+^ T cells has been shown to increase HCC burden in mice. Furthermore, increased infiltration of CD8^+^ T cells in tumors has been reported to be associated with better immunotherapy response in HCC.^3^ To investigate the effect of ICB in NASH-HCC, Pfister et al.^1^ first examined the T cells composition across three NASH mice models (CD-HFD, WD-HFD, and hURI-tetOFF^hep^) and one normal diet control with single-cell RNA-sequencing (scRNA-seq), flow cytometry, and mass spectrometry. They observed that the frequencies of CD8^+^PD1^+^ cells were specifically increased in NASH-affected livers. Furthermore, RNA-seq data indicated that the CD8^+^PD1^+^ T cells had features of effector (*Gzmk* and *Gzmm*), exhaustion (*Pdcd1*), and tissue residency (*Cxcr6*). Given the high infiltration of CD8^+^PD1^+^ T cells in NASH, their re-activation in NASH-HCC by anti-PD1 treatment is expected to generate meaningful responses in tumor suppression.

Surprisingly, none of the liver tumors in NASH-HCC mice regressed upon anti-PD1 treatment, while tumors in non-NASH HCC cancer models were shrunk by identical regimens. Moreover, liver fibrosis was exacerbated after anti-PD1 treatment in NASH-HCC models. These findings indicated that anti-PD1 treatment failed to reinvigorate exhausted CD8^+^PD1^+^ T cells to execute anti-tumor surveillance; instead, anti-PD1 endowed this T cell subset with enhanced effector signature and tissue-damaging potential. They further verified the function of CD8^+^PD1^+^ T cells in the precancerous NASH mice model. In non-cancerous mice with NASH, anti-PD1 treatment could lead to increased liver CD8^+^PD1^+^ T cells, aggravated liver damage, and increased tumor incidence. On the contrary, depletion of CD8^+^ T cells could lead to alleviated tissue damage and decreased tumor incidence. Based on these observations, they hypothesize that CD8^+^PD1^+^ T cell-mediated tissue damage may underlie the promotion of hepatocarcinogenesis upon anti-PD1 treatment in NASH models.

To explore the underlying mechanism for CD8^+^PD1^+^ T cells to trigger the development of HCC, Pfister et al.^1^ adopted scRNA-seq and high-parametric flow cytometry to further characterize CD8^+^PD1^+^ T cells after anti-PD1 treatment. They found that anti-PD1 treatment increased the abundance of resident-like CD8^+^PD1^+^ T cells with features of hyperactive effector and exhaustion features. Hepatic resident-like CD8^+^ T cells are reported to trigger auto-aggression in the pathogenesis of NASH.^4^ CD8^+^PD1^+^ T cells are also dysfunctional for effective anti-tumor surveillance and are mediators of NASH-HCC progression. Notably, a sub-population CD8^+^PD1^+^TNF^+^ T cells were significantly enriched upon anti-PD1 treatment. The combination of anti-PD1 and anti-TNF therapy ameliorated tissue damage, liver inflammation and prevented tumorigenesis compared to single anti-PD1 therapy. These results indicated that CD8^+^PD1^+^ T cells drove hepatic inflammation and subsequent liver cancer in a TNF-dependent manner upon anti-PD1 immunotherapy.

Pfister et al.^1^ next questioned if their results in animal models can be extrapolated to human NASH. They profiled CD8^+^PD1^+^ T cells in liver biopsies patients with non-alcoholic fatty liver disease (NAFLD) or NASH and identified similar transcriptional features and auto-aggressive characteristics in human NAFLD and NASH. Furthermore, a meta-analysis including three phase III clinical trials of anti-PD(L)1 immunotherapy comprising of over 1600 patients revealed that immunotherapy did not improve survival in patients with non-viral HCC. Focusing on the effect of anti-PD(L)1 immunotherapy in NAFLD-HCC, the authors investigated two additional cohorts which enrolled small groups of NAFLD-HCC patients. Consistent with their hypothesis, they found a shortened overall survival after immunotherapy of NAFLD-HCC patients as compared with patients with other malignancies. Collectively, both pre-clinical and clinical data indicate that anti-PD(L)1 immunotherapy may not confer beneficial effects in NAFLD-HCC.

This timely study signals a word of caution in the accelerated approvals of ICB therapy for advanced, Sorafenib-pretreated HCC patients, regardless of the disease etiology. As the etiology of HCC is significantly different across different populations, this might contribute to differential response to ICB therapy. For example, in the IMbrave150 with the combination of Atezolizumab plus Bevacizumab in HCC, the median overall survival of 19.2 months was achieved in the whole population. Of note, updated subgroup analysis of Chinese patients, which have higher HBV infection rates, showed superior survival (24 months).^5^ These results are in line with the present study, which suggests immunotherapy in viral HCC. Further subgroup analysis of viral and non-viral HCC in the IMbrave150 cohort should be considered to comprehensively investigate the immunotherapy efficacy in HCC.

This study has significant clinical ramifications for immunotherapy in NASH patients. First it implies a potentially worse outcome for NASH-HCC patients receiving ICB therapy. Furthermore, the ongoing studies on ICB therapy as adjuvant therapy after surgical resection or ablation of NASH-HCC should be re-evaluated with caution, as ICB therapy might increase the risk of cancer recurrence in this subgroup of patients. Finally, it is unclear if NASH as an underlying condition might impact ICB therapy efficacy in non-HCC tumors (e.g., lung cancer, melanoma, and colorectal cancer). More studies are required to evaluate if ICB therapy in these patients might increase the risk for liver damage and HCC occurrence in these patients.

**Fig. 1:**
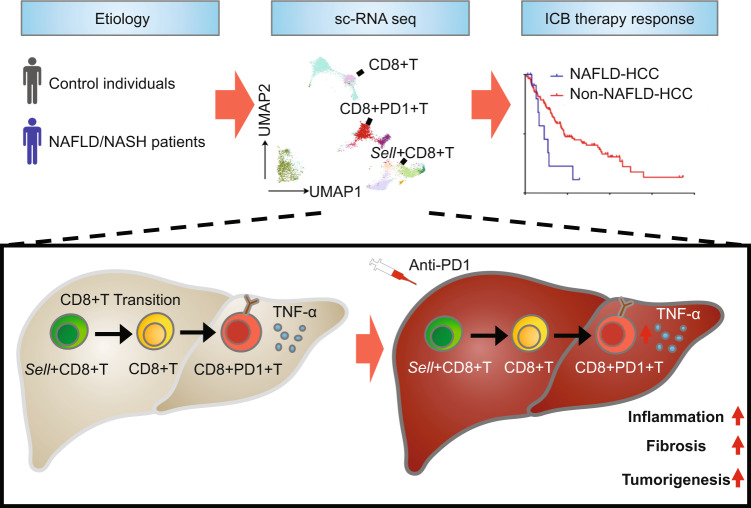
Anti-PD1 immunotherapy promotes NALFD-associated HCC. sc-RNA seq analysis of NAFLD/NASH patients identified the hepatic-resident-like and exhausted CD8^+^PD1^+^ T cells. Mechanistic studies in animal models reveal progressive accumulation of unconventionally activated CD8^+^PD1^+^ T cells in the NASH liver, and anti-PD1 treatment promotes CD8^+^PD1^+^ T cells population and stimulates them to generate TNF-α, leading to increased inflammation, fibrosis, and tumorigenesis. Survival analysis in HCC patients shows worse outcome in NAFLD-HCC patients receiving anti-PD1 immunotherapy

In summary, the elegant work by Pfister et al.^1^ demonstrated pro-tumorigenic property of CD8^+^PD1^+^ T cells, and their involvement in anti-PD1 immunotherapy triggered NASH-HCC preclinical models. Besides, they found CD8^+^PD1^+^ T cells with similar traits in clinical samples and suggested the lack of anti-PD(L)1 immunotherapy response in NAFLD-HCC patients. These findings may have a significant impact on clinical practice in HCC immunotherapy. In the future, extensive work of larger cohorts of immunotherapy treated NASH/HCC patients is urgently needed to verify the rational patient stratification for optimal therapy response (Fig. 1).
